# Prognostic value of the combination of volume, massiveness and fragmentation parameters measured on baseline FDG pet in high-burden follicular lymphoma

**DOI:** 10.1038/s41598-024-58412-0

**Published:** 2024-04-05

**Authors:** S. Draye-Carbonnier, V. Camus, S. Becker, D. Tonnelet, E. Lévêque, A. Zduniak, F. Jardin, H. Tilly, P. Vera, P. Decazes

**Affiliations:** 1 Department of Nuclear Medicine, Centre Henri Becquerel, Rouen, France; 2 Department of Hematology, Centre Henri Becquerel, Rouen, France; 3 INSERM U1245, Université de Rouen, IRIB, Rouen, France; 4 QuantIF-LITIS (EA 4108-FR CNRS 3638), Faculty of Medicine, University of Rouen, Rouen, France; 5 Department of Statistics and Clinical Research Unit, Centre Henri Becquerel, Rouen, France

**Keywords:** ^18^F-FDG PET/CT, Follicular lymphoma, Prognosis, Tumor burden, Radiomics, Non-hodgkin lymphoma, Cancer

## Abstract

The prognostic value of radiomic quantitative features measured on pre-treatment ^18^F-FDG PET/CT was investigated in patients with follicular lymphoma (FL). We conducted a retrospective study of 126 FL patients (grade 1-3a) diagnosed between 2006 and 2020. A dozen of PET/CT-derived features were extracted via a software (*Oncometer3D*) from baseline ^18^F-FDG PET/CT images. The receiver operating characteristic (ROC) curve, Kaplan–Meier method and Cox analysis were used to assess the prognostic factors for progression of disease within 24 months (POD24) and progression-free survival at 24 months. Four different clusters were identified among the twelve PET parameters analyzed: activity, tumor burden, fragmentation-massiveness and dispersion. On ROC analyses, TMTV, the total metabolic tumor volume, had the highest AUC (0.734) followed by medPCD, the median distance between the centroid of the tumors and their periphery (AUC: 0.733). Patients with high TMTV (HR = 4.341; *p* < 0.001), high Tumor Volume Surface Ratio (TVSR) (HR = 3.204; *p* < 0.003) and high medPCD (HR = 4.507; *p* < 0.001) had significantly worse prognosis in both Kaplan–Meier and Cox univariate analyses. Furthermore, a synergistic effect was observed in Kaplan–Meier and Cox analyses combining these three PET/CT-derived parameters (HR = 12.562; *p* < 0.001). Having two or three high parameters among TMTV, TVSR and medPCD was able to predict POD24 status with a specificity of 68% and a sensitivity of 75%. TMTV, TVSR and baseline medPCD are strong prognostic factors in FL and their combination better predicts disease prognosis.

## Introduction

Follicular Lymphoma (FL) is an indolent CD20 + Non-Hodgkin Lymphoma (NHL) and the most common low-grade B-cell lymphoma, accounting for 25% of all the NHL in Western countries^1^. and occurs predominantly at an advanced age^2^.

FL is a non-curable indolent disease with frequent relapses but with median survival time exceeding 10 years^3^. The clinical course can be very heterogeneous at many levels with a wide variety of presentations, histological appearances, clinical behaviors and responses to therapy.

Treatment varies depending on the stage and clinical presentation. Therefore, accurate staging is crucial for appropriate management. Whether the disease is localized or in an advanced stage, numerous frontline treatment options can be proposed and range from watchful waiting, single-agent rituximab (R) to external radiation therapy for low tumor burden^4^ or immuno-chemotherapy for high tumor burden according to the Groupe d’Etude des Lymphomes Folliculaires (GELF) criteria^5^. Since the use of anti-CD20 monoclonal antibodies, primarily Rituximab, combined with chemotherapy and used as maintenance therapy for two years thereafter, the overall survival rate of FL patients has remarkably improved^6^.

Despite the improvements in long-term disease control, about 20% of patients affected by FL ultimately experience treatment failure and progression of disease within 24 months (POD24) from the time of diagnosis with a 5-years OS rate of only 50% for them^7^. POD24 is the main prognostic factor affecting the outcome of FL patients^8,9^. Furthermore, histological transformation of FL occurs in 5–10% of patients with a 2% increased risk per year after the diagnosis^10^ (usually DLBCL) resulting in a poor prognosis^11^. Therefore, early identification at diagnosis of patients who will present an early-relapse or progression is needed to guide therapeutic strategy, as at present time no biomarker or tool exist to anticipate POD24 + patients at diagnosis.

Despite its indolent biology, FL is FDG-avid in more than 95% of the cases, regardless of tumor grade^12–16^. Recent guidelines recommend ^18^F-FDG Positron Emission Tomography/Computed Tomography (PET/CT) in clinical practice as a standard of care in FL for diagnosis, baseline staging, suspicion of transformation, best suitable re-biopsy site in the context of relapse and at time of response assessment (gold standard)^17–21^.

Effectively, PET/CT is more sensitive and specific than standard computed tomography (CT) scans or MRI^22,23^ with better detection of all disease sites, particularly in identifying extra-nodal disease, altering stage assignation and changes management by up-staging or down-staging in around 20% of FL patients^24,25^. Furthermore, CT is unable to distinguish between viable tumor and fibrosis in the post-therapy residual masses^26–29^.

Numerous studies suggested higher values of quantitative maximum standardized uptake value (SUVmax)^30^, total metabolic tumor volume (TMTV) parameter^31–34^ and qualitative Deauville index are considered to be associated with inferior survival at baseline ^18^F-FDG PET/CT evaluation in FL. The prognostic value of TMTV obtained from baseline ^18^F-FDG PET/CT has been recently reported in patients with various subtypes of lymphomas ^35–42^*.*

With the development of the radiomics, more complex quantitative parameters than TMTV, which more precisely evaluate the tumor burden, can be extracted from ^18^F-FDG PET/CT data and analyzed, such as tumor’s massiveness-fragmentation, dispersion or activity^43,44^. There is increasing evidence that these ^18^F-FDG PET/CT quantitative parameters may also have predictive value in FL^45–47^, but a definitive consensus has not been achieved yet.

FL patients with a high risk of treatment failure or early-relapse cannot be easily identified by the classic prognostic clinical indicators such as the FLIPI-1^48^ or FLIPI-2^49^. Thus, there is a need for new reliable prognostic biomarkers to better select high-risk patient categories that can benefit from personalized, risk-adapted, treatment strategies, shortly after diagnosis.

The aim of the present study was to measure ^18^F-FDG PET/CT-derived quantitative parameters in newly diagnosed patients with high tumor burden FL and investigate their potential role, alone or in combination, as predictive factors for POD24 at baseline imaging.

## Methods

### Patients

We carried out a retrospective observational monocentric cohort study called “LYMFOTEP” in the Nuclear Medicine department and the Hematology department of Henri Becquerel Cancer Centre, Rouen, France. Research protocol was approved by the Institutional Review Board of Henri Becquerel Centre (no. 2005B). Patients were informed about the use of anonymized data for research and their right to oppose this use. Written informed consent was waived because of the retrospective nature of the study. The study respected the ethical principles of the 2008 Helsinki Declaration.

The inclusion criteria were as follows: (1) all patients were aged 18 years or older with a (2) histological based diagnosis of follicular lymphoma (3) grade 1-3a in accordance with the World Health Organization (WHO) classification, between 2006 and 2020, (4) treated with first line immuno-chemotherapy including anti-CD20 monoclonal antibodies for high tumor burden according to GELF criteria with a (5) mandatory baseline ^18^F-FDG PET/CT examination, performed prior to initiation of therapy and (6) patient’s non-opposition statement.

Exclusion criteria were as follows: (1) patients with other malignant tumors, (2) known histological transformation at baseline, (3) aggressive B-cell lymphoma, (4) FL grade 3b according to the WHO classification and (5) no available ^18^F-FDG PET/CT at baseline.

### Clinical data

Baseline patient’s disease characteristics (FLIPI, POD24) and survival data were obtained from internal medical records. Clinical data obtained from all patients included the following information: gender, age at disease onset, disease characteristics, LDH, Hemoglobin, ECOG score and treatment regimens.

### PET acquisition and interpretation

All patients underwent ^18^F-FDG PET/CT with acquisitions performed according to the Society of Nuclear Medicine and Molecular Imaging (SNMMI) and the European Association of Nuclear Medicine (EANM) guidelines^50^. Patients were instructed to fast for at least six hours before ^18^F-FDG injection. The radiopharmaceuticals (^18^F-FDG) were supplied by CURIUM**®** or PETNET**®** and manufactured in accordance with Good Manufacturing Practices and the European Pharmacopoeia. Injection was not performed unless glucose blood level was below < 1.8 g/L. ^18^F-FDG intravenous injected activity was around 2.5–4 MBq/kg, as a function of the PET/CT device used: Biograph 16 (Siemens Medical Solutions, Knoxville, TN, USA), Biograph 40 (Siemens Medical Solutions, Knoxville, TN, USA), Discovery 710 (GE Healthcare, Milwaukee, WI, USA) or Biograph Vision-600 (Siemens Medical Solutions, Knoxville, TN, USA) with a maximum activity of 450 MBq, after 30 min of rest. Scans were acquired approximately 60 min (± 5 min) after injection. CT scans for attenuation correction and anatomic localization were acquired from the mid-thigh toward the base of the skull in most cases and whole-body acquisition was realized in others, with 100 to 120 kV and 100–150 mAs (based on patient’s weight), in helical mode. Contrast media injection was not performed. Images were reconstructed with validated and commercially available iterative algorithms (Ordered-Subset Expectation Maximization iterative reconstruction). The PET systems were normalized daily and the calibration coefficient was validated if the day-to-day variation remained below 0.3%. The global quantification, from the dose calibrator to the imaging system, was measured internally on a quarterly basis and double checked by the EARL’s quality assurance program.

^18^F-FDG PET/CT data was anonymized and collected in DICOM format. All the data was then retrospectively reviewed and integrated in an eCRF. Quantitative PET parameters and measurements were performed and extracted by a trained nuclear physician, unaware of clinical outcome or patient characteristics. Data was analyzed using the plug-in PET/CT viewer for FIJI (ImajeJ), a freeware from the Beth Israel Deaconess Medical Centre, Division of Nuclear Medicine and Molecular Imaging^51,52^. 41% of the maximum standardized uptake value (SUVmax) was applied as a threshold^53^. First, segmentation was performed automatically using the software and was then checked visually to confirm inclusion of only pathological lesions. A manual verification and adaptation was then performed, if needed. Lesions sites were determined according to visual assessment with ^18^F-FDG PET/CT images scaled to a fixed SUV display and color table. Each hypermetabolic focus suspected of malignant disease localization was segmented on fused PET/CT images. Segmentations of the hypermetabolic lymph nodes, spleen, bone and other pathological foci were saved separately.

Lesions considered as pathological were identified visually as areas of increased uptake outside areas of physiological uptake (e.g. brain, heart, urinary system etc.). For the bone marrow and spleen involvement, only the focal uptakes were included. However, in case of a diffuse and intense spleen uptake, the whole spleen was included if its SUV was greater than 150% of the liver background^54^.

### Radiomic parameters

A total of twelve quantitative 3-D PET/CT-derived parameters were then extracted with the software *Oncometer3D v1.0*^43^ https://www.researchgate.net/publication/378659728_Oncometer3D_10], an exhaustive description and graphical representation of these PET parameters are available in Supplemental Data.

### Statistical analysis

Continuous variables are reported as mean ± SD with minimal and maximal values. Categorical variables are expressed as numbers and percentages.

The relationship between the different PET metrics was characterized by the Spearman’s rank correlation coefficient. A correlogram was represented thanks to corrplot R function. It corresponded to correlation matrix between the twelve PET/CT parameters associated with the significance of each correlation coefficient.

POD24 (progression of disease within 24 months), a binary variable, was defined as disease progression within 24 months after first line immuno-chemotherapy, while non-POD24 was defined as the absence progression within 24 months of first-line therapy. Receiver operating characteristic (ROC) analysis for POD24 was used to determine the optimal cut-off value for each feature by maximizing the product of sensitivity and specificity*.* Sensitivity and specificity were calculated for that suitable cut-off. The area under the curve (AUC) was also calculated.

Progression-Free Survival (PFS), a continuous variable, was defined from treatment initiation to disease progression, death for any reason or relapse up to 24 months.

Overall Survival (OS) was defined as the time from treatment initiation to the date of death by any cause.

Survival curves were obtained with the Kaplan–Meier method. Quantitative PET/CT variable was dichotomized according to the established cut-off from ROC analysis. For each binary variable, comparison of survival curves between categories was assessed by log-rank test. Cox Proportional Hazards Model was performed to evaluate the relationship between study variables and survival rates. Statistical significance was set at a two-tailed «*p*» value < 0.05.

## Results

### Patients’ characteristics and outcomes

**Figure 1:**
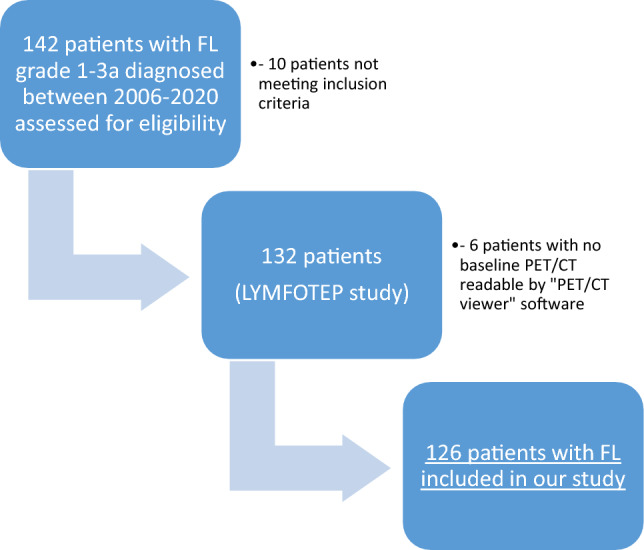
Flow chart of patient selection for the study.

One hundred and twenty-six patients extracted from the “LYMFOTEP” study with previously untreated FL, considered as “high” tumor burden according to GELF criteria and available baseline ^18^F-FDG PET/CT were included in the study (see flowchart in Fig. 1). Description of the population with clinical characteristics is available in Table 1. Patients (65 males and 61 females) had a median age of 61 (range 35–88) and the vast majority (86.5%) received immuno-chemotherapy with R-CHOP regimen. The median follow-up was 120 months.

**Table 1:** Baseline characteristics of follicular lymphoma patients (n = 126).

Characteristics	n = 126
Age (years)	
Mean (± SD)	60.6 (± 9.9)
Min; max	35.0; 88.0
Gender	
Female	61 (48.4%)
Male	65 (51.6%)
FLIPI	
Low	12 (9.6%)
High	62 (49.6%)
Intermediate	51 (40.8%)
Treatment regimen	
R-CHOP	109 (86.5%)
R2	9 (7.1%)
RB	5 (4.0%)
G-CHOP	2 (1.6%)
GB	1 (0.8%)
POD24	
+	28 (22%)
−	98 (78%)
LDH	
Mean (± SD)	147.4 (± 141.8)
Min; max	55.0; 904.0
Hemoglobin	
Mean (± SD)	13.4 (± 1.6)
Min; max	8.9; 16.3
ECOG score	
0	92 (73.0%)
1	24 (19.0%)
2	8 (6.3%)
3	1 (0.8%)
4	1 (0.8%)
Maintenance therapy	
Yes	80 (63.5%)
No	46 (36.5%)
Antibody therapy maintenance	
Rituximab	78 (97.5%)
Obinutuzumab	2 (2.5%)
Perfusions numbers	
Mean (± SD)	10.8 (± 3.0)
Min; max	1; 13
Length of maintenance treatment (days)	
Mean (± SD)	576.1 (± 187.5)
Min; max	0; 923

*FLIPI* Follicular Lymphoma International Prognostic Index, *R-CHOP* rituximab cyclophosphamide doxorubicin vincristine prednisone, *R2* rituximab lenalidomide, *RB* rituximab bendamustine, *G-CHOP* obinutuzumab cyclophosphamide doxorubicin vincristine prednisone, *GB* obinutuzumab bendamustine, *POD24* Progression Of Disease within 24 months, *LDH?* Lactate Dehydrogenase, *ECOG* Eastern Cooperative Oncology Group.

Mean values of the twelve baseline ^18^F-FDG PET/CT tumor’s features evaluated are reported in Table 2. Patients were separated between two groups according to their POD24 status. A first group: POD24- defined by POD24 = 0 and including 98 patients (77.8%) and a second group: POD24 + defined by POD24 = 1 including 28 patients (22.2%). Distributions were significantly different between POD24 + and POD24-for TMTV (*p* < 0.001), TLG (*p* < 0.001), TVSR (*p* = 0.009), TMTS (*p* < 0.001), TumBB (*p* = 0.038), medEDGE (*p* = 0.003), medPCD (*p* < 0.001) and itErosion (*p* = 0.003).

**Table 2:** Description of the twelve parameters extracted from baseline PET/CT.

Parameters Mean (± SD) min; max	Study population (n = 126)	POD24- (n = 98)	POD24 + (n = 28)	*p*-value (Wilcoxon test)
SUVmax	14.2 (± 7.0) 5.4; 57.1	14.3 (± 7.4) 5.4; 57.1	14.0 (± 5.6) 5.7; 27.9	0.718
TMTV (cm^3^)	867.8 (± 731.0) 68.7; 3002.9	723.6 (± 637.3) 68.7; 2989.0	1372.6 (± 821.6) 135.0; 3002.9	** < 0.001**
TLG	3857.3 (± 3819.5) 192.5; 20905.0	3247.3 (± 3530.1) 192.5; 20905.0	5992.6 (± 4085.5) 679.3; 15851.9	** < 0.001**
Dmax (mm)	570.4 (± 198.3) 81.2; 960.8	558.5 (± 203.1) 81.2; 960.8	612.1 (± 177.5) 160.9; 822.5	0.15
SUVmean	4.3 (± 1.8) 2.1; 10.6	4.2 (± 1.8) 2.1; 10.6	4.4 (± 1.7) 2.4; 8.4	0.548
TVSR (mm)	5.2 (± 1.6) 2.9; 11.5	5.1 (± 1.6) 2.9; 11.5	5.8 (± 1.6) 3.6; 9.2	**0.009**
TMTS (cm^2^)	1619.9 (± 1193.1) 142.7; 5039.7	1401.6 (± 1029.4) 142.7; 4759.3	2384.2 (± 1415.6) 234.9; 5039.7	** < 0.001**
TumBB (cm^3^)	22571.7 (± 16054.2) 227.4; 72072.0	20809.1 (± 15112.6) 227.4; 56594.9	28740.9 (± 17943.6) 689.5; 72072.0	**0.038**
nROI	13.2 (± 13.2) 1.0; 122.0	11.7 (± 8.2) 1.0; 45.0	18.5 (± 23.0) 2.0; 122.0	0.179
medEDGE (mm)	37.2 (± 12.6) 19.9; 81.2	35.8 (± 12.3) 19.9; 81.2	42.0 (± 13.0) 24.6; 72.6	**0.003**
medPCD (mm)	37.6 (± 16.2) 14.4; 91.3	35.1 (± 15.5) 14.4; 91.3	46.5 (± 15.7) 22.0; 82.1	** < 0.001**
itErosion	2.8 (± 0.8) 1.8; 6.3	2.7 (± 0.8) 1.8; 6.3	3.1 (± 0.9) 1.9; 5.0	**0.003**

Significant values are in [bold].

*SUVmax* maximum standardized uptake value, *TMTV* total metabolic tumour volume, *TLG* total lesion glycolysis, *Dmax* largest distance between two lesions, *SUVmean* mean standardized uptake value, *TVSR* tumour volume surface ratio, *TMTS* total metabolic tumour surface, *TumBB* tumour bounding box, *nROI* number of regions of interest, *medEDGE* median edge distance, *medPCD* median distance between the centroid of the tumour and its periphery, *itErosion* iterative erosion.

The 10-years OS was 78.4% for the whole population in our study.

### ^18^F-FDG PET/CT metrics and correlations

**Figure 2:**
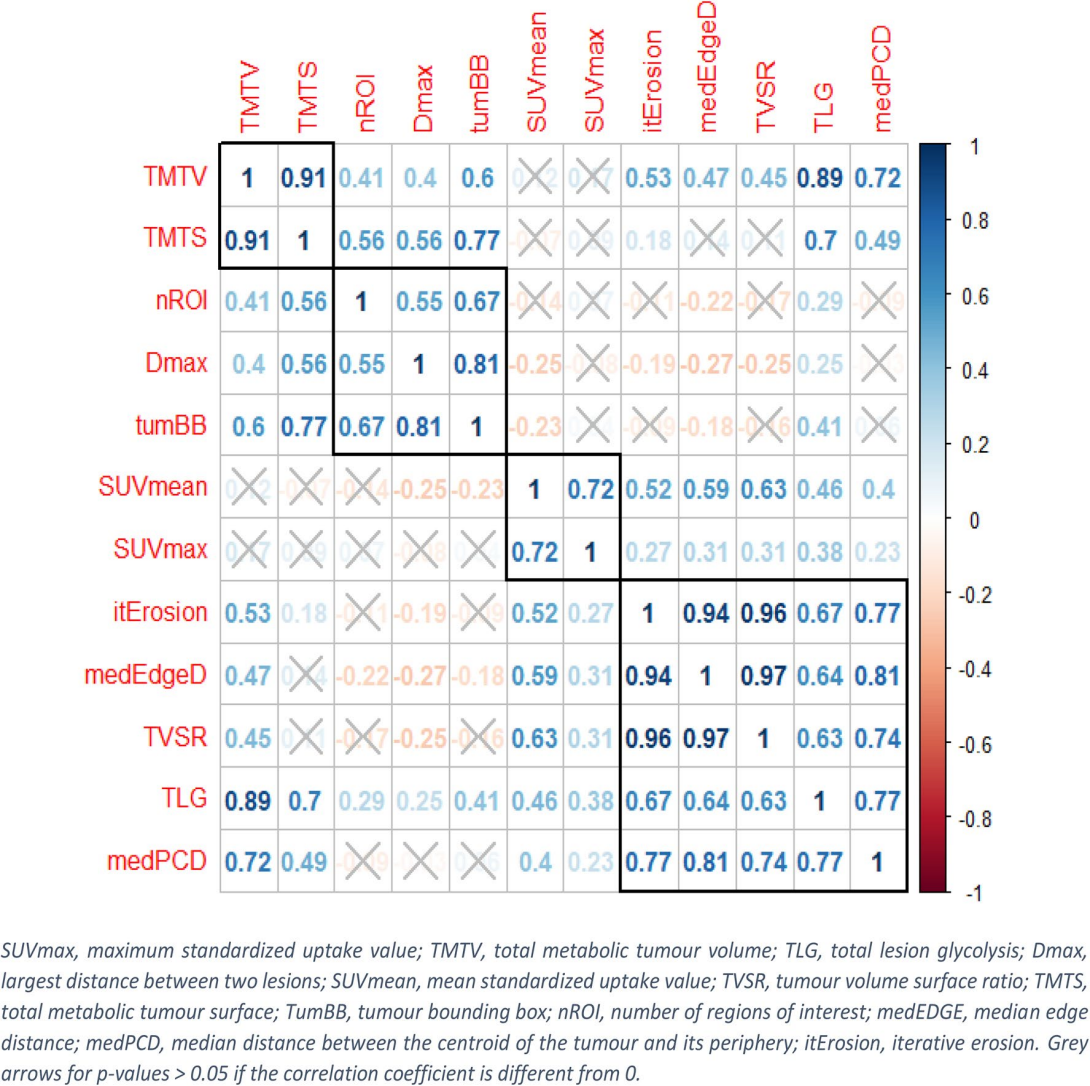
Correlogram between the twelve PET/CT parameters with numeric values. SUVmax, maximum standardized uptake value; TMTV, total metabolic tumour volume; TLG, total lesion glycolysis; Dmax, largest distance between two lesions; SUVmean, mean standardized uptake value; TVSR, tumour volume surface ratio; TMTS, total metabolic tumour surface; TumBB, tumour bounding box; nROI, number of regions of interest; medEDGE, median edge distance; medPCD, median distance between the centroid of the tumour and its periphery; itErosion, iterative erosion. Grey arrows for *p*-values > 0.05 if the correlation coefficient is different from 0.

As visible in the correlogram (Fig. 2), four different clusters, combining highly correlated parameters among the twelve PET parameters analyzed, could be identified:

- Activity (SUVmax; SUVmean),
- Tumor burden (TMTV; TMTS),
- Massiveness/fragmentation (TVSR; medPCD; medEDGE; itErosion),
- Dispersion (Dmax; TumBB; nROI)

Interestingly, TMTS was highly correlated with TMTV (ρ = 0.91) and therefore had low added prognostic value compared to TMTV, while by contrast, TVSR (ratio between TMTV and TMTS) was rather uncorrelated with TMTV (ρ = 0.45) and could have therefore an added prognostic value. In addition, TLG was the only parameter significantly correlated with all others, including parameters belonging to different clusters, it was, in particular, highly correlated to TMTV (ρ = 0.89).

### ROC curve analysis

The ROC curve analysis for POD24 is showed in Table 3 where optimal cut-off, AUC, performance parameters and repartition of patients below/above cut-off were presented. TMTV had the highest area under the curve (AUC = 0.734), followed by medPCD (AUC = 0.733) and TLG (AUC = 0.715). TVSR, medEDGE, itErosion, TMTS and TumBB had also AUC significantly different from 0.5.

**Table 3:** Diagnostic performances of the 12 PET/CT-derived parameters for POD24 using a ROC analysis.

	AUC [95% CI]	Cut-off value	Sensitivity	Specificity	Number of patients below cut-off (%)	Number of patients above cut-off (%)
SUVmax	0.528 [0.404; 0.652]	12.286	0.571	0.557	67 (53.2%)	59 (46.8%)
TMTV	**0.734 [0.621; 0.848]**	1195.248	0.607	0.806	90 (71.4%)	36 (28.6%)
TLG	**0.715 [0.6; 0.83]**	3223.187	0.75	0.704	76 (60.3%)	50 (39.7%)
Dmax	0.59 [0.468; 0.711]	725.328	0.429	0.806	95 (75.4%)	31 (24.6%)
SUVmean	0.462 [0.343; 0.582]	3.715	0.5	0.51	61 (48.4%)	65 (51.6%)
TMTS	**0.707 [0.59; 0.825]**	1894.169	0.643	0.735	82 (65.1%)	44 (34.9%)
TVSR	**0.663 [0.554; 0.772]**	4.854	0.714	0.612	67 (53.2%)	59 (46.8%)
TumBB	**0.629 [0.507; 0.752]**	26,208	0.571	0.684	79 (62.7%)	47 (37.3%)
nROI	0.583 [0.453; 0.713]	17	0.429	0.786	93 (73.8%)	33 (26.2%)
medEDGE	**0.683 [0.582; 0.784]**	33.641	0.821	0.571	60 (47.6%)	66 (52.4%)
medPCD	**0.733 [0.636; 0.83]**	36.932	0.786	0.663	71 (56.3%)	55 (43.7%)
itErosion	**0.683 [0.576; 0.789]**	2.385	0.857	0.5	56 (44.4%)	70 (55.6%)

Significant values are in [bold].

*SUVmax* maximum standardized uptake value, *TMTV* total metabolic tumour volume, *TLG* total lesion glycolysis, *Dmax* largest distance between two lesions, *SUVmean* mean standardized uptake value, *TVSR* tumour volume surface ratio, *TMTS* total metabolic tumour surface, *TumBB* tumour bounding box, *nROI* number of regions of interest, *medEDGE* median edge distance, *medPCD* median distance between the centroid of the tumour and its periphery, *itErosion* iterative erosion.

### Kaplan–Meier survival analysis

**Figure 3:**
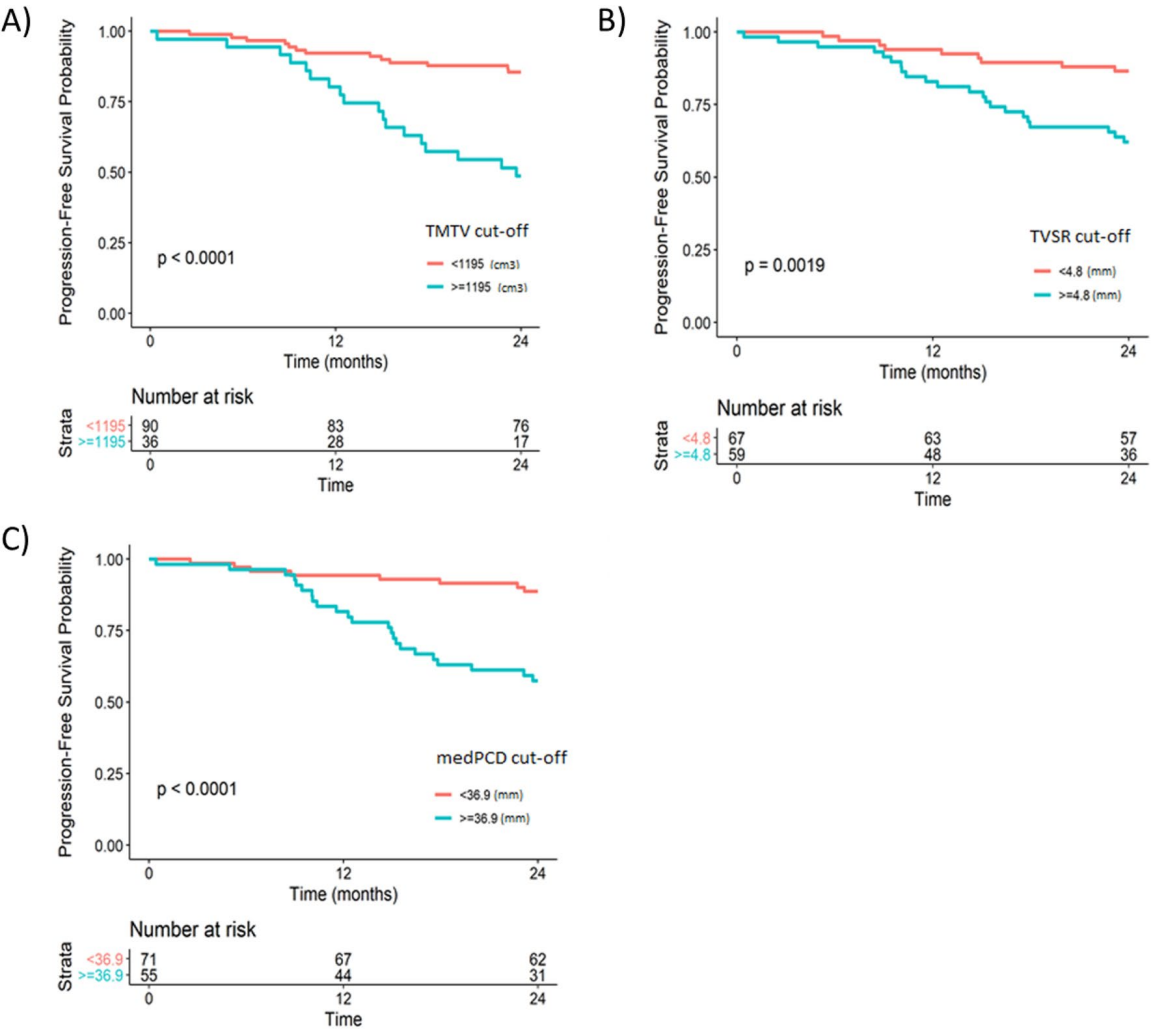
Kaplan–Meier survival analysis for PFS at 24 months according to the TMTV (**A**), TVSR (**B**) and medPCD (**C**).

A Kaplan–Meier survival analysis was performed according to the cut-off values of the ROC curves for POD24. Burden parameters (TMTV, TMTS and TLG) and fragmentation parameters (TVSR, medPCD, medEDGE and itErosion) had statistically significant log-rank tests (all *p*-values < 0.001). Graphical representations for TMTV (*p* < 0.001), TVSR (*p* = 0.0019) and medPCD (*p* < 0.001) are represented in Fig. 3.

### Cox univariate analysis

Cox univariate analyses are presented in Table 4 and Tables 1 and 3. in Supp. Data. The univariate analysis showed that neither FLIPI score, nor male sex were significantly associated with PFS censored at 24 months (FLIPI-High: *p* = 0.3; FLIPI-Intermediate: *p* = 0.9; Male sex: *p* = 0.2) or uncensored PFS.

**Table 4:** Univariate cox analysis for PFS at 24 months.

Variables	Log rank test (p-value)	HR	Lower bound 95% CI	Upper bound 95% CI	*p*-value
SUVmax (≥ 12.3, ref: < 12.3)	0.5	1.276	0.631	2.58	0.5
TMTV (≥ 1195, ref: < 1195)	**1e−05**	**4.341**	2.121	8.884	**6e−05**
TLG (≥ 3223, ref: < 3223)	**2e−05**	**4.628**	2.127	10.07	**1e−04**
Dmax (≥ 725, ref: < 725)	0.05	2.062	1.001	4.251	0.05
SUVmean (≥ 3.7, ref: < 3.7)	0.7	1.177	0.58	2.388	0.7
TVSR (≥ 4.8, ref: < 4.8)	**0.002**	**3.204**	1.474	6.963	**0.003**
TMTS (≥ 1894, ref: < 1894)	**4e−04**	**3.426**	1.661	7.067	**9e−04**
TumBB (≥ 26,208, ref: < 26,208)	0.08	1.858	0.918	3.761	0.08
nROI (≥ 17, ref: < 17)	0.08	1.88	0.912	3.876	0.09
medEDGE (≥ 33.6, ref: < 33.6)	**4e−04**	**4.325**	1.773	10.551	**0.001**
medPCD (≥ 36.9, ref: < 36.9)	**6e−05**	**4.507**	2.013	10.095	**3e−04**
itErosion (≥ 2.4, ref: < 2.4)	**0.006**	**3.043**	1.31	7.067	**0.01**
FLIPI—High (ref: Low)	0.1	2.289	0.535	9.795	0.3
FLIPI—Intermediate (ref: Low)		1.07	0.231	4.954	0.9
Gender (Male, ref: Female)	0.2	1.618	0.785	3.334	0.2
Combined Score: TMTV + TVSR − High (ref: Low)	**2e−05**	**3.507**	1.235	9.958	**0.02**

Significant values are in [bold].

In contrast, high burden parameters (TMTV, TLG, TMTS) and high fragmentation parameters (TVSR, medEDGE, medPCD and itErosion) were significantly associated with less favorable survival rates for PFS and OS with the highest Hazard Ratio (HR) for TLG (HR = 4.628; 95% CI 2.13–10.07; *p* < 0.001), followed by medPCD (HR = 4.507; 95% CI 2.01–10.10; *p* < 0.001) and TMTV (HR = 4.341; 95% CI 2.12–8.88; *p* < 0.001) for POD24.

### Combination of parameters: TMTV, TVSR and medPCD

Due to the high correlation observed between some of the parameters with significant statistical value in univariate analysis, a combination of parameters from different clusters appeared to be more appropriate than a multivariate analysis including all significant parameters. A combination of three parameters (TMTV, TVSR and medPCD) was then performed.

Patients could be divided into four sub-groups according to the threshold obtained in ROC analyses: “0” for no high parameter among the three, “1” for only one high parameter, “2” for two high parameters and “3” for all three high parameters.

Distribution of the patients according to that classification and POD24 status is presented in Table 5. Groups “0” and “1” represented 67 patients over the 98 patients POD24- while groups “2” and “3” represented 21 patients over the 28 patients POD24+ , thus a specificity of 68% and sensitivity of 75% for this categorization to determine POD24 status.

**Table 5:** Description of combined score evaluating TMTV, TVSR and medPCD according to POD24 status.

	Study population	POD24−	POD24+
Number of high parameters (TMTV; TVSR; medPCD)			
N	126	98	28
0	53 (42%)	51 (52%)	2 (7%)
1	21 (17%)	16 (16%)	5 (18%)
2	27 (21%)	18 (19%)	9 (32%)
3	25 (20%)	13 (13%)	12 (43%)

*TMTV* total metabolic tumour volume, *TVSR* tumour volume surface ratio, medPCD median distance between the centroid of the tumour and its periphery.

**Figure 4:**
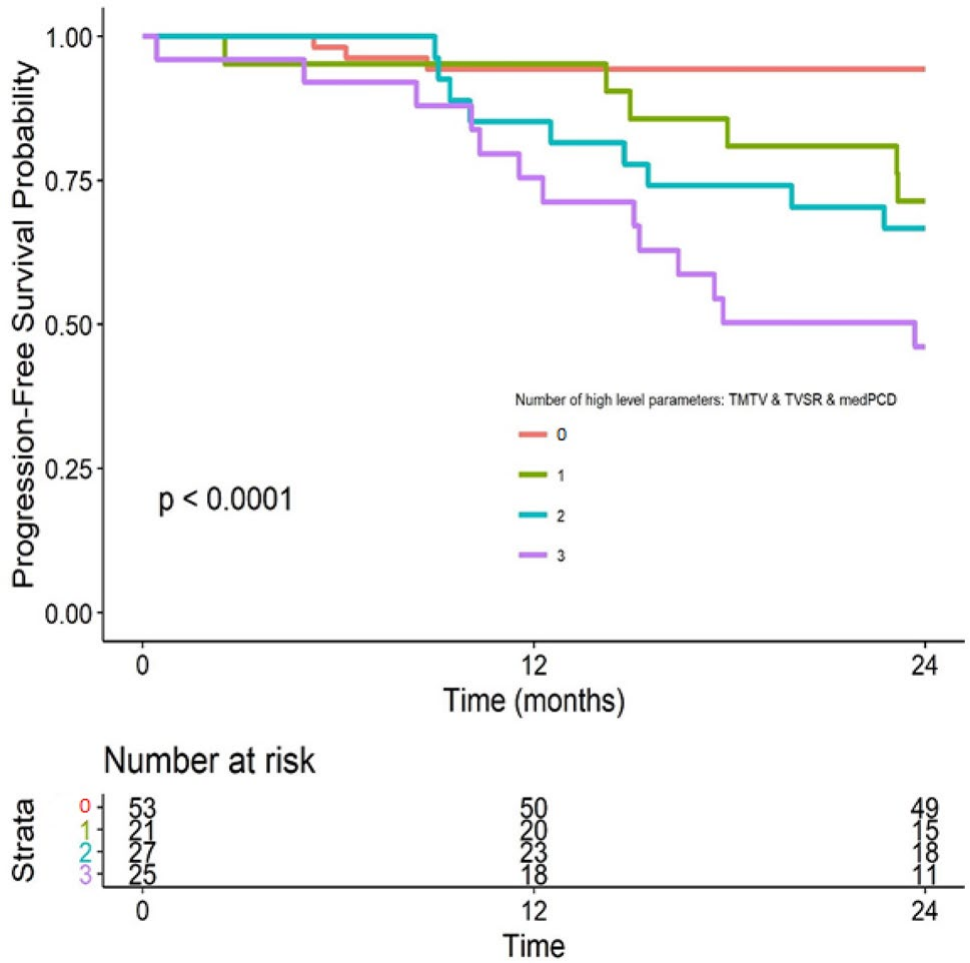
Kaplan–Meier survival analysis for PFS at 24 months according to the combination score (TMTV + TVSR + medPCD).

Kaplan–Meier survival curves are available in Fig. 4 and Fig. 2 and 3 in Supp. Data and show significantly different survival curves according to the number of high parameters. The smallest probability of survival was observed for the patients group combining three high level parameters.

Consistently, in Cox analyses (Table 6 and Tables 2 and 4 in Supp. Data), patients with 3 high level parameters had a significantly worse PFS at 24 months (HR = 12.562; 95% CI 3.57–44.20; p < 0.001) than patients with 2 high level parameters (HR = 6.75; 95% CI 1.83–24.95; *p* = 0.004) or patients with only 1 high level parameter (HR = 5.36; 95% CI 1.34–21.44; *p* = 0.02), showing the synergistic effect of the combination of these 3 PET parameters.

**Table 6:** Cox analysis for combined score for PFS at 24 months (TMTV + TVSR + medPCD).

	Log-rank test (*p*-value)	HR	95% CI	*p*-value
Number of high-level parameters (TMTV & TVSR & medPCD): 1 (ref: 0)	4e-05	5.36	1.34–21.439	0.02
Number of high-level parameters (TMTV & TVSR & medPCD): 2 (ref: 0)		6.75	1.826–24.95	0.004
Number of high-level parameters (TMTV & TVSR & medPCD): 3 (ref: 0)		12.562	3.57–44.201	8e−05

*TMTV* total metabolic tumour volume, *TVSR* tumour volume surface ratio, *medPCD* median distance between the centroid of the tumour and its periphery.

**Figure 5:**
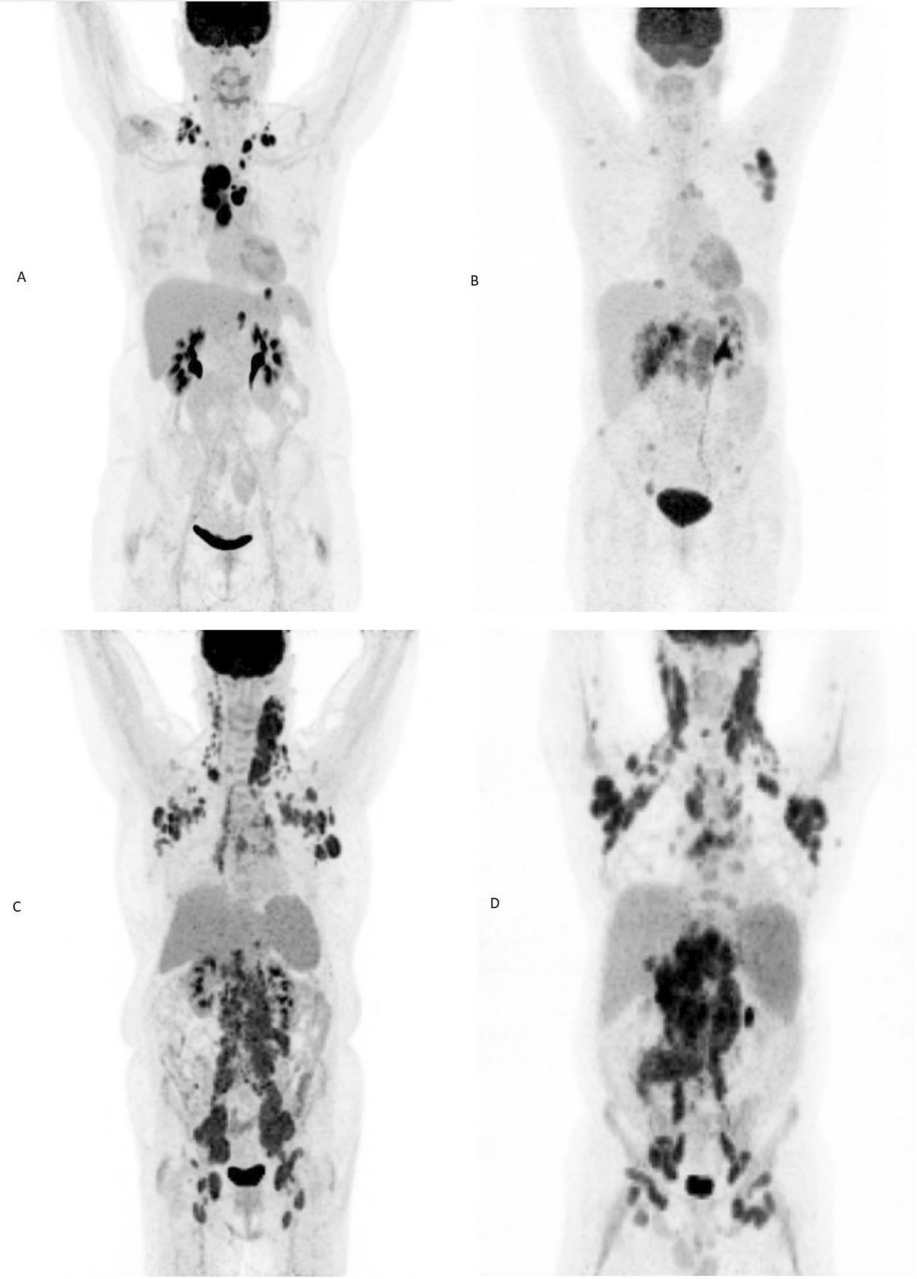
Maximal intensity projection (MIP) images of 1-3A follicular lymphoma patients group 0 (A: TMTV 202.7 cm^3^; TVSR 4.3 mm; medPCD 18.1 mm), group 1 (B: TMTV 211.5 cm^3^; TVSR 5.0 mm; medPCD 29 mm), group 2 (C: TMTV 1440.8 cm^3^; TVSR 4.6 mm; medPCD 49.7 mm) and group 3 (D: TMTV 2823.1 cm^3^; TVSR 5.8 mm; medPCD 39.5 mm), according to the combination score (TMTV; TVSR; medPCD).

Examples of the four sub-groups of the newly established scoring system using maximal intensity projection (MIP) on PET images are shown in Fig. 5.

## Discussion

The prognostic value of PET/CT-derived parameters has been investigated in various lymphoma entities, in addition to the standard qualitative visual analysis (Deauville five-point scale). The current study evaluates the association and the prognostic impact of different PET/CT biomarkers such as TMTV, TVSR and medPCD from baseline ^18^F-FDG PET/CT in a cohort of more than a hundred FL patients with high tumor burden according to GELF criteria, mainly treated with R-CHOP immuno-chemotherapy. Our study confirms the strong and significant prognostic value of tumoral features and their help, notably by combining them, in the early identification of FL patients with a high risk of early progression of disease within 24 months after first-line treatment.

The primary strength of our study is the novel finding that ^18^F-FDG PET/CT parameters such as TVSR and medPCD have a high prognostic value for POD24 in FL patients. Thus, we established a prognostic stratification model based on three features (TMTV, TVSR and medPCD) and the creation of four different risk groups. The results indicates that PET/CT-derived features might be helpful in the prognostic evaluation and treatment personalization of FL patients. These three PET parameters represent complementary and distinct aspects of the hematology malignancy, which may explain their additional prognostic power.

Considering the parameters separately, TMTV, as in our observations, has already been linked to an unfavorable prognostic in high tumor burden FL, regardless of the segmentation method used but also for many, if not all, types of lymphoma^31,34^. The optimal TMTV cut-off found for PFS in our study was 533.5 cm^3^ (AUC = 0.63), similar to the 510 cm^3^ (AUC = 0.7) found by Meignan et al.^31^ and Cottereau et al.^33^ for PFS, who also used a 41% SUVmax threshold (median TMTV in our study was 606 vs 297 cm^3^). However, the optimal TMTV cut-off in our results relative to POD24 was 1195 cm^3^. This result could have been due to the high total tumor burden with GELF criteria of the patients included in our study (50% of patients with high burden tumor with a FLIPI score 3–5) as well as the differences in treatments.

**Figure 6:**
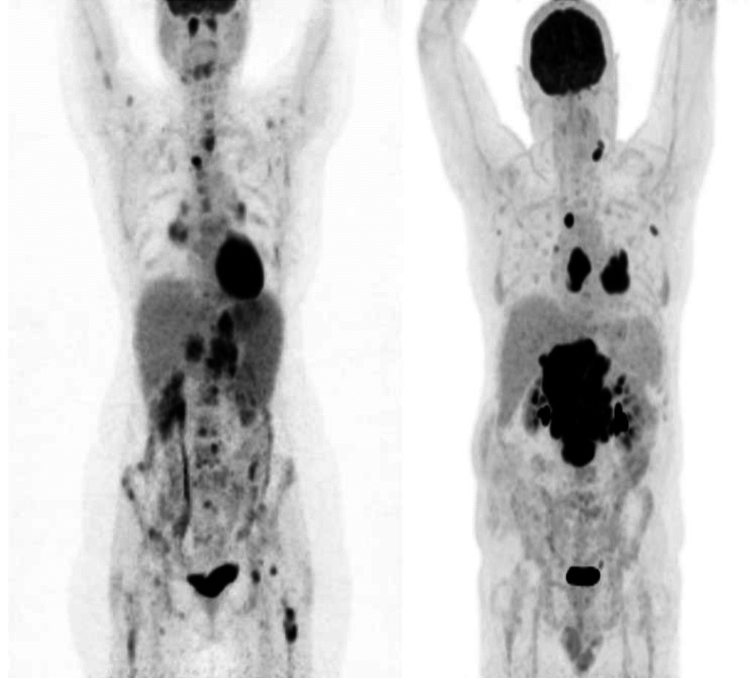
(Left) Example MIP image of patient with high TMTV (1353 cm3) and low TVSR (5.1 mm), in favor of a fragmented tumor. (Right) Example MIP image of patient with both high TMTV (1346 cm3) and high TVSR (9.2 mm), in favor of a massive tumor.

TVSR is the ratio of two parameters: TMTV and TMTS and represents the tumor fragmentation. To illustrate this parameter, Fig. 6 gives the 2D representation of two patients with almost similar TMTV (1353 cm3 and 1346 cm3) but with different TVSR (5.1 mm and 9.2 mm respectively). The first patient, who had a more fragmented tumor, survived more than ten years after the beginning of treatment (OS: 129.45 months) while the other patient survived less than four years (OS: 45.34 months). In our study, a high value suggesting a massive tumor had a significantly worse prognosis. Our findings are consistent with the results previously published in DLBCL patients and reinforce the prognostic impact of the combination of TMTV and TVSR^44^.

Complementary results are observed with medPCD which is the median distance between the centroid of the tumor and its periphery and describes tumor’s massiveness with an unfavorable prognosis in massive tumors. TMTV and medPCD may be linked to a worse response to treatment due to less tissue-penetration of anti-cancer drugs. Therefore, more intensive chemo-immunotherapy might be considered for FL patients with high baseline TVSR and medPCD.

High baseline TLG has recently been showed to be a strong prognostic factor in FL^45–47^. We also highlighted its prognostic value. However, we observed that this parameter was too highly correlated with the totality of the PET parameters analyzed and not enough discriminant, being probably at the crossroads of all parameters studied. In our opinion, it could be more interesting to combine three different and relatively uncorrelated PET parameters exploring different aspects of the multi-site tumor rather than one parameter. However, because of its "central" character, it is possible that this parameter has an interesting alternative value, especially to describe the disease in a more generic way than TMTV.

In addition, dissemination feature Dmax was not significantly linked with survival in our study (*p* = 0.05) contrary to data found in other lymphomas such as DLBCL^37^ or Hodgkin lymphoma^55^. Only a tendency was found, showing that this promising parameter may not be associated with survival in this particular lymphoma entity.

Regarding the segmentation method used in this study, The SUV41% method, recommended by the European Association of Nuclear Medicine (EANM) for delineating lesions in lymphoma studies, uses threshold for volume contouring, where voxels included in the lesion volume have an SUV of at least 41% of the hottest voxel in that lesion^56^. The main weakness of this method is the risk of an underestimation of true lesion volumes if the FDG uptake is very heterogeneous. Furthermore, it can overestimate the TMTV of lesions with low SUVmax value. Different thresholds can be applied depending on the type of tumor, the radiotracer used, and the specific clinical context. Therefore, an SUV threshold of 4 is sometimes used as a fixed cutoff point to differentiate between benign and malignant lesions^57^, as it is generally above the range for benign conditions. However, this higher threshold may not be as sensitive in detecting all metabolically active lesions, especially in diseases like follicular lymphoma where lesions can have variable metabolic activity, sometimes low. Thus, further studies are still needed to determine whether the reference method should change for follicular lymphoma.

However, in our cohort of more than a hundred patients, only two of them had a SUVmax < 6 and no significant difference was made for SUVmax between FL patients with high or low TMTV. Measurements in routine practice appeared limited owing to the multiplicity of segmentation methods and its time-consuming nature in daily practice. Modern softwares allow to obtain volume computation in a few seconds and only leaves the exclusion of non-pathological regions which have been erroneously selected by the software as a task to the physician to improve efficiency. As a result, TMTV, TVSR and medPCD measurements could now become possible in clinical routine, especially with the help of fully automated or even machine-learning platforms^58^.

It should be noted that most of the PET/CT-derived parameters analyzed in this study are geometrical parameters, describing shape. Contrary to the majority of radiomic textural features, these parameters are robust and less sensitive to differences in PET/CT devices or even reconstruction algorithms^59–61^. Therefore, harmonization of data extracted from different PET/CT scans are unnecessary given the nature of the parameters explored. Furthermore, the PET/CT parameters examined in this study are easily understandable from a biological point of view.

From a mathematical perspective, if lymphoma tumors were perfectly spherical, TVSR and medPCD should be perfectly correlated because they are radius dependent. If we observed a limited correlation between these two parameters (ρ = 0.74), it is precisely because lymphoma cannot be considered as spheres.

Concerning the survival, we chose not to study OS given the known prolonged OS in FL patients and the small number of events (Supplemental Data; Fig. 1). For this pathology, the POD24 is considered as a surrogate marker for OS in clinical trials^62,63^. Combination of TMTV, TVSR and medPCD at baseline may help physicians to anticipate POD24 + and to propose alternative, risk-adapted, treatment strategies in this high-risk population with unmet medical need in order to improve patient’s outcomes.

Finally, our study has some limitations, such as the single center retrospective nature, the lack of a validation cohort, the existence of potential selection bias and results may not be extrapolated to patients with low tumor burden according to GELF criteria. Consequently, large-scale prospective multi-center studies are worth performing to confirm our conclusions.

## Conclusion

In conclusion, TMTV, representing the total tumor burden, TVSR, describing the tumor fragmentation and medPCD, illustrating the tumor massiveness, measured on baseline ^18^F-FDG PET/CT are strong prognostic factors in FL patients that require treatment.

Combination of TMTV, TVSR and medPCD is promising and has a synergistic effect. A prognostic scoring system consisting of these three PET-derived parameters could be useful to improve risk stratification at baseline imaging and help to identify a group of high-risk patients, which may benefit from more personalized treatment strategies.

### Supplementary Information

Supplementary Information.
