# Supplementary material for: Prognostic value of the combination of volume, massiveness and fragmentation parameters measured on baseline FDG pet in high-burden follicular lymphoma
Source: Sci Rep. 2024 Apr 5;14:8033. doi: 10.1038/s41598-024-58412-0 (PMC10997640; doi:10.1038/s41598-024-58412-0)
Supplement: Supplementary file 1 — Supplementary Information. [file 41598_2024_58412_MOESM1_ESM.docx]

# Supplemental data

***Figure 1****: Overall survival curve censored at 10 years.*


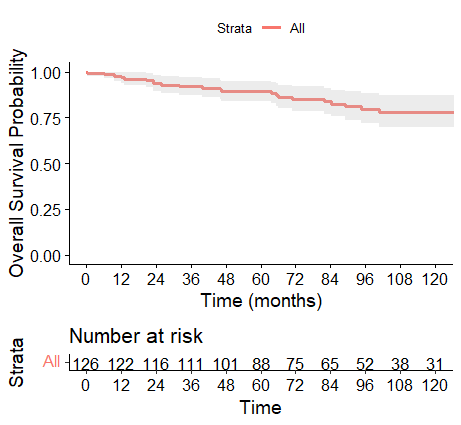


**Figure 2:** Kaplan-Meier survival analysis for uncensored PFS according to the combination score (TMTV + TVSR + medPCD).


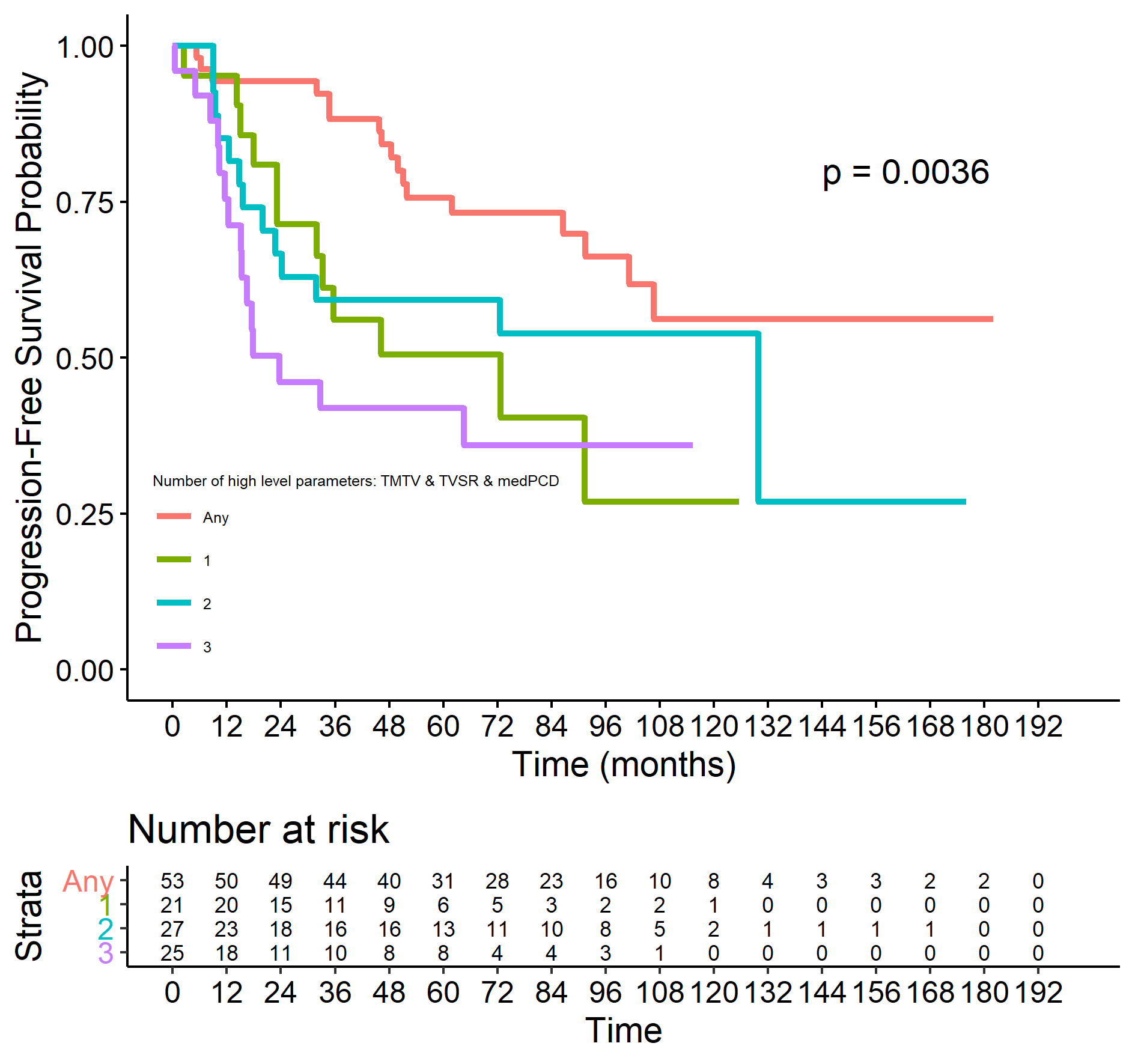


**Figure 3:** Kaplan-Meier survival analysis for OS according to the combination score (TMTV + TVSR + medPCD).


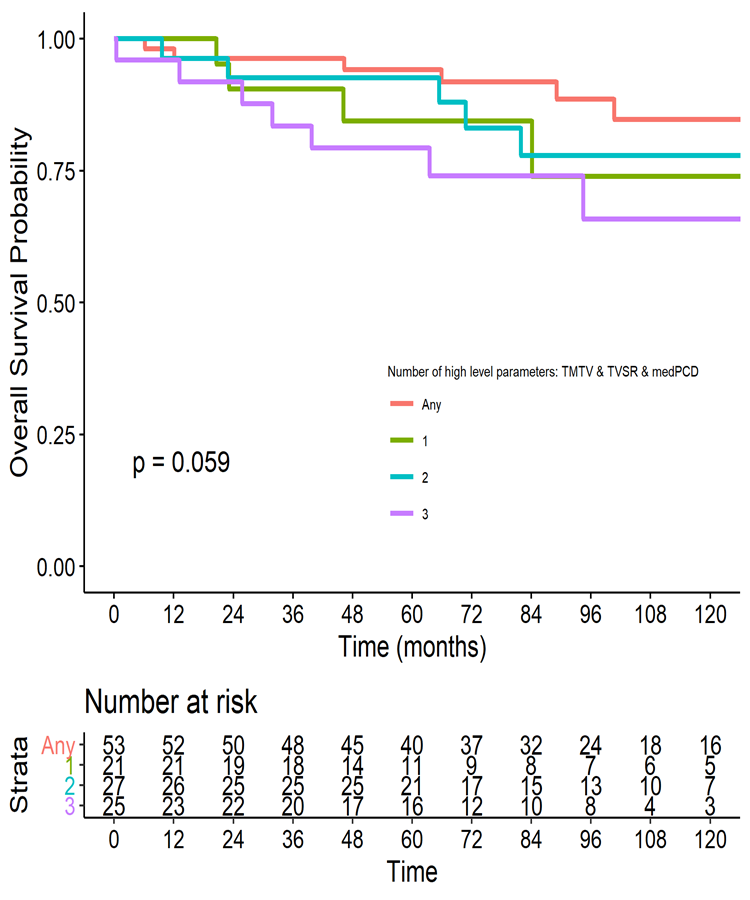


**Table 1:** Univariate Cox analysis for uncensored PFS.

| **Variables** | **Log rank test**  **(p-value)** | **HR** | **Lower bound 95% CI** | **Upper bound 95% CI** | **p-value** |
| --- | --- | --- | --- | --- | --- |
| SUVmax (>=12.3, ref: <12.3) | 1 | 0.996 | 0.589 | 1.684 | 1 |
| TMTV (>=1195, ref: <1195) | **0.003** | **2.249** | 1.303 | 3.881 | **0.04** |
| TLG (>=3223, ref: <3223) | **0.006** | **2.061** | 1.219 | 3.484 | **0.007** |
| Dmax (>=725, ref: <725) | 0.08 | 1.645 | 0.93 | 2.911 | 0.09 |
| SUVmean (>=3.7, ref: <3.7) | 0.6 | 0.858 | 0.509 | 1.446 | 0.6 |
| TVSR (>=4.8, ref: <4.8) | **0.01** | **1.921** | 1.136 | 3.247 | **0.01** |
| TMTS (>=1894, ref: <1894) | **0.005** | **2.079** | 1.231 | 3.512 | **0.006** |
| TumBB (>=26208, ref: <26208) | **0.02** | **1.867** | 1.105 | 3.155 | **0.02** |
| nROI (>=17, ref: <17) | **0.04** | **1.782** | 1.023 | 3.104 | 0.04 |
| medEDGE (>=33.6, ref: <33.6) | **0.02** | **1.887** | 1.105 | 3.222 | 0.02 |
| medPCD (>=36.9, ref: <36.9) | **0.01** | **1.916** | 1.137 | 3.228 | 0.01 |
| itErosion (>=2.4, ref: <2.4) | **0.05** | **1.715** | 0.999 | 2.944 | **0.05** |
| FLIPI - High (ref: Low) | 0.1 | 1.57 | 0.611 | 4.033 | 0.3 |
| FLIPI - Intermediate (ref: Low) |  | 0.894 | 0.335 | 2.387 | 0.8 |
| Gender (Male, ref: Female) | **0.001** | **2.432** | 1.392 | 4.249 | **0.002** |
| Treatment : R-CHOP (ref : other) | 0.6 | 1.205 | 0.545 | 2.662 | 0.6 |

*SUVmax, maximum* *standardized uptake value; TMTV, total metabolic tumour volume; TLG, total lesion glycolysis; Dmax, largest distance between two lesions; SUVmean, mean standardized uptake value; TVSR, tumour volume surface ratio; TMTS, total metabolic tumour surface; TumBB, tumour bounding box; nROI, number of regions of interest; medEDGE, median edge distance; medPCD, median distance between the centroid of the tumour and its periphery; itErosion,* *iterative erosion.*

**Table 2**: Cox analysis for combined score for uncensored PFS (TMTV + TVSR + medPCD).

|  | Log-rank test (p-value) | HR | 95 % CI | p-value |
| --- | --- | --- | --- | --- |
| Number of high-level parameters (TMTV & TVSR & medPCD): **1**  (ref: 0) | **0.004** | **2.49** | 1.18 – 5.255 | **0.02** |
| Number of high-level parameters (TMTV & TVSR & medPCD): **2**  (ref: 0) |  | 1.836 | 0.891 – 3.784 | 0.1 |
| Number of high-level parameters (TMTV & TVSR & medPCD): **3**  (ref: 0) |  | **3.386** | 1.675 – 6.844 | **7e-04** |

*TMTV, total metabolic tumour volume; TVSR, tumour volume surface ratio; medPCD, median distance between the centroid of the tumour and its periphery*

**Table 3:** Univariate Cox analysis for uncensored OS.

| **Variables** | **Log rank test**  **(p-value)** | **HR** | **Lower bound 95% CI** | **Upper bound 95% CI** | **p-value** |
| --- | --- | --- | --- | --- | --- |
| SUVmax (>=12.3, ref: <12.3) | 1 | 0.996 | 0.589 | 1.684 | 1 |
| TMTV (>=1195, ref: <1195) | **0.003** | **2.249** | 1.303 | 3.881 | **0.004** |
| TLG (>=3223, ref: <3223) | **0.006** | **2.061** | 1.219 | 3.484 | **0.007** |
| Dmax (>=725, ref: <725) | 0.08 | 1.645 | 0.93 | 2.911 | 0.09 |
| SUVmean (>=3.7, ref: <3.7) | 0.6 | 0.858 | 0.509 | 1.446 | 0.6 |
| TVSR (>=4.8, ref: <4.8) | **0.01** | **1.921** | 1.136 | 3.247 | **0.01** |
| TMTS (>=1894, ref: <1894) | **0.005** | **2.079** | 1.231 | 3.512 | **0.006** |
| TumBB (>=26208, ref: <26208) | **0.02** | **1.867** | 1.105 | 3.155 | **0.02** |
| nROI (>=17, ref: <17) | **0.04** | **1.782** | 1.023 | 3.104 | **0.04** |
| medEDGE (>=33.6, ref: <33.6) | **0.02** | **1.887** | 1.105 | 3.222 | **0.02** |
| medPCD (>=36.9, ref: <36.9) | **0.01** | **1.916** | 1.137 | 3.228 | **0.01** |
| itErosion (>=2.4, ref: <2.4) | 0.05 | 1.715 | 0.999 | 2.944 | 0.05 |
| FLIPI - High (ref: Low) | 0.1 | 1.57 | 0.611 | 4.033 | 0.3 |
| FLIPI - Intermediate (ref: Low) |  | 0.894 | 0.335 | 2.387 | 0.8 |
| Gender (Male, ref: Female) | **0.001** | **2.432** | 1.392 | 4.249 | **0.002** |

*SUVmax, maximum standardized uptake value; TMTV, total metabolic tumour volume; TLG, total lesion glycolysis; Dmax, largest distance between two lesions; SUVmean, mean standardized uptake value; TVSR, tumour volume surface ratio; TMTS, total metabolic tumour surface; TumBB, tumour bounding box; nROI, number of regions of interest; medEDGE, median edge distance; medPCD, median distance between the centroid of the tumour and its periphery; itErosion,* *iterative erosion.*

**Table 4**: Cox analysis for combined score for uncensored OS (TMTV + TVSR + medPCD).

|  | Log-rank test (p-value) | HR | 95 % CI | p-value |
| --- | --- | --- | --- | --- |
| Number of high-level parameters (TMTV & TVSR & medPCD): **1**  (ref: 0) | **0.004** | **2.49** | 1.18 – 5.255 | **0.02** |
| Number of high-level parameters (TMTV & TVSR & medPCD): **2**  (ref: 0) |  | 1.836 | 0.891 – 3.784 | 0.1 |
| Number of high-level parameters (TMTV & TVSR & medPCD): **3**  (ref: 0) |  | **3.386** | 1.675 – 6.844 | **7e-04** |

*TMTV, total metabolic tumour volume; TVSR, tumour volume surface ratio; medPCD, median distance between the centroid of the tumour and its periphery*

*Exhaustive description of the twelve PET parameters studied.*

- The Standardized Uptake Value (SUV), the activity concentration normalized to the injected activity, divided by the total body weight. The highest SUV in a volume of interest (VOI) is the SUVmax. The highest SUVmax over all lesions of the patient was reported.

- The SUVmean, the mean value of SUV measured in all the tumors.

- The Total Metabolic Tumor Volume (TMTV), which refers to the tumor’s metabolically active volume. It is the sum of the metabolic volumes of all nodal and extra-nodal lesions.

- The Total Lesion Glycolysis (TLG), obtained by multiplying the total metabolic activity of each lymphoma lesion (MTV) by the SUVmean.

- The Total Metabolic Tumor Surface (TMTS), the sum of the metabolic surfaces of all the tumors (tumor–healthy tissue interface).

- The Tumor Volume Surface Ratio (TVSR), the ratio between the TMTV and the TMTS, describing the tumor fragmentation. A high value would indicate a massive tumor while a low value would correspond to a more fragmented tumor.

- The Dmax, the distance between the two lesions that are the furthest apart, deduced from the 3D coordinates of the baseline TMTV and captures the dissemination/spread of the disease.

- The volume of the bounding box including the tumors (TumBB), representing the volume of tumor dispersion.

- The number of regions of interest (nROI), describing the number of unique tumors on the whole examination.

- The iterative erosion (itErosion), corresponding to the number of erosions necessitated to remove tumors from the images (pixels of 2x2x2 mm).

- The median edge distance (medEDGE) corresponding to the median distance between the opposite edges of the tumors.

- The median distance between the centroid of the tumors and their periphery (medPCD), representing tumor’s massiveness.

For visual purpose, we propose a planar representation of the twelve different PET parameters extracted:


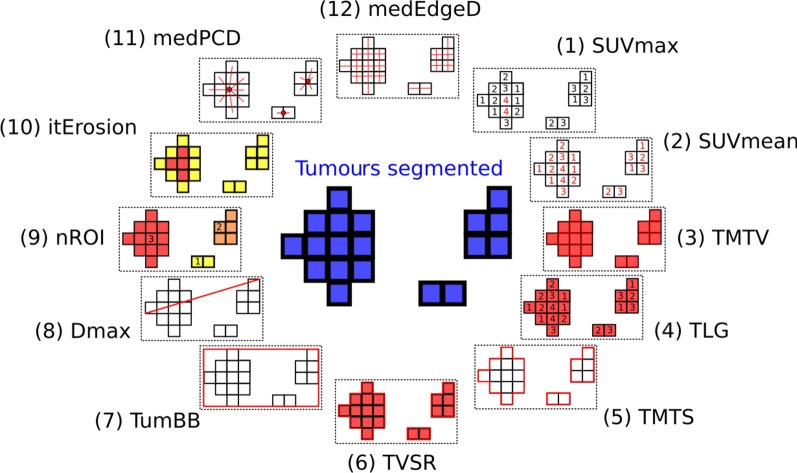


Representation of the twelve different PET parameters measured by the software **Oncometer3D** and analysing burden, activity, dispersion, fragmentation and massiveness of the lymphoma. For graphical purpose, a planar representation of these 3D parameters is shown. Decazes et al 2020. All rights reserved.
